# Housing type and risk of malaria among under-five children in Nigeria: evidence from the malaria indicator survey

**DOI:** 10.1186/s12936-018-2463-6

**Published:** 2018-08-28

**Authors:** Oyewale M. Morakinyo, Folusho M. Balogun, Adeniyi F. Fagbamigbe

**Affiliations:** 1 Department of Environmental Health Sciences, Faculty of Public Health, College of Medicine, University of Ibadan, Ibadan, Nigeria; 2 Institute of Child Health, College of Medicine, University of Ibadan, Ibadan, Nigeria; 3 Department of Epidemiology and Medical Statistics, Faculty of Public Health, College of Medicine, University of Ibadan, Ibadan, Nigeria

**Keywords:** Housing type, Malaria, Under-five, Rapid diagnostic test, Microscopy

## Abstract

**Background:**

Malaria remains one of the major causes of morbidity and mortality among under-five (U5) children in Nigeria. Though different environmental factors have been assessed to influence the distribution and transmission of malaria vectors, there is a dearth of information on how housing type may influence malaria transmission among U5 children in Nigeria. This study assessed the relationship between housing type and malaria prevalence among U5s in Nigeria.

**Methods:**

A cross-sectional analysis of the nationally representative 2015 Nigeria malaria indicator survey data was done. A representative sample of 8148 households in 329 clusters was selected for the survey. Children aged 6–59 months in the selected households were tested for anaemia and malaria using the rapid diagnostic test (RDT) and the microscopy. Data were analysed using descriptive statistics, Pearson Chi square (χ^2^) and logistic regression models at 5% level of significance.

**Results:**

The odds of malaria infection was significantly higher among older children aged 24–59 months (aOR = 4.8, CI 2.13–10.99, p < 0.001), and children who lived in houses built completely with unimproved materials (aOR = 1.4, CI 1.08–1.80, p = 0.01). Other predictors of malaria infection include living in a rural area (aOR = 1.5, CI 1.25–1.91, p = 0.01), ever slept under a long-lasting insecticide-treated net (aOR = 1.1, CI 0.26–4.79, p = 0.89) and in a room not sprayed with insecticide (aOR = 1.2, CI 0.64–2.31, p = 0.56). Children who were malaria positive showed a higher prevalence of severe anaemia on RDT (87.6%) and Microscopy (67.4%) than those who were not anaemic (RDT = 31.6%, Microscopy = 12.9%).

**Conclusions:**

Non-improved housing predicted malaria infection among U5s in Nigeria. Improved housing is a promising means to support a more integrated and sustainable approach to malaria prevention. Education of the Nigerian people on the role of improved housing on malaria protection and empowerment of the public to adopt improved housing as well as overall enlightenment on ways to prevent malaria infection can help to augment the current malaria control measures among U5 children.

## Background

Malaria, an ancient threat to human health, remains a primary cause of morbidity and mortality globally [1, 2]. The 2016 world malaria report indicated that 212 million cases and 429,000 deaths were recorded in 2015 [3]. Two-third of this deaths occurred among under-five (U5) children in Africa [3]. About 25% of malaria burden in Africa [4] occurred in Nigeria where 60% of outpatients’ visits and 30% of hospital admissions are as a result of malaria [5].

Most often, patients present with non-specific symptoms, such as fever, rigors, and chills. Severe malaria develops mainly among children and may manifest as extreme weakness, impaired consciousness, severe anaemia, respiratory distress, convulsions, and hypoglycaemia, among other symptoms [6]. The occurrence of long-term neurological sequelae from severe malaria [7], subtle developmental and cognitive impairments as a result of both severe and uncomplicated episodes [8] have been reported in children. Moreover, anaemia is one of the complications that accompany malaria infections and it plays a significant role in its morbidity and mortality [9].

However, recent epidemiological reports have signified a reduction in the global burden of malaria [2, 10]. This has been attributed to the up-scaling up of malaria prevention and control interventions, including the use of long-lasting insecticide-treated nets (LLINs), indoor residual spraying (IRS), intermittent preventive therapy for pregnant women (IPTp), improved use of malaria rapid diagnostic tests (RDTs) and effective treatments using artemisinin-based combination therapy (ACT) [10, 11].

Nevertheless, the rising problem of insecticide-resistant mosquitoes is hindering the usefulness of some of these interventions, with some species of mosquitoes in Western and Southern Africa being resistant to the currently available classes of insecticide in public health [12–14]. Researchers have pointed out the likelihood of resistant mosquitoes surviving up to a thousand times in the minimal concentration of insecticide that normally kills susceptible mosquitoes [14–16]. Instances of blood-fed mosquitoes present in LLINs or resting on sprayed walls have also been reported [14–16]. Continuous use of these interventions suggests that lessons have not been learnt from the problems of insecticide resistance which occurred in the 50s and 60s during the global malaria eradication programme [17–19].

Different malaria control programmes have been introduced, and these include the WHO Global Technical Strategy for Malaria 2016–2030 (GTS), the complementary Roll Back Malaria (RBM), and the multi-sectoral Action and Investment to Defeat Malaria 2016–2030 (AIM). The goals of these programmes are to achieve a 90% decline in mortality and case incidence of malaria, complete eradication from 35 countries by 2030 and avert the re-introduction into malaria-free areas. Although these are laudable initiatives, there is an obvious and urgent need to pay more attention to the newer interventions which are stated in the guidelines of these programmes as this will complement the previous reliance on the use of LLINs and IRS [20, 21].

The role of housing in reducing the burden of malaria resonated throughout the AIM framework [11, 20]. There is a growing interest in the desire to use ‘modern’ housing as an intervention against malaria burden as recently illustrated in the new RBM work stream on malaria and housing [11]. Housing is an important determinant of health and quality of life [22]. The design of a house contributes to the incidence of *Plasmodium falciparum* infection [23, 24]. Researchers have reported that living in an improved housing played a significant role in the eradication of malaria in the United States and its reduction in Europe [25, 26]. It played a vital role in controlling human exposure to mosquitoes and vector-borne diseases [27]. A 42% lower odds of malaria infection and a 54–65% lower incidence of clinical malaria have been reported among occupants of improved housing than unimproved houses in The Gambia [11].

Although the possibility for an association between housing type and malaria incidence has been established, the evidence base remains sparse compared to the evidence associated with the use of LLINS or IRS. In Nigeria, there is a dearth of information on how housing type may influence malaria transmission across locations, regions, and household wealth among children U5. The objectives of this study were to describe the pattern of malaria parasite infection among U5 children and to determine the relationship between housing characteristics and malaria infection among this age group in Nigeria. The significance of the current study lies in its ability to provide an evidence-based recommendation on the importance of improved housing to accelerating the progress towards the control and elimination of malaria on a large scale in Nigeria. It will also influence the formulation of policy that will promote the design of healthy houses which can contribute to the reduction in the threat of malaria among children U5 years of age.

## Methods

### Study area

This study was conducted in Nigeria, a federal republic in West Africa. Administratively, it is divided into 36 states and a federal capital territory (FCT). These states are further classified into six geopolitical zones: North-West, North-Central, North-East, South-East, South-West, and South–South. In each of the geopolitical zones, the people are similar in nature and unique in their ways of life including health-related characteristics and housing types.

### Data source

This study utilized data from the 2015 Nigeria Malaria Indicator Survey (NMIS). The 2015 NMIS was designed to provide useful information on malaria indicators and prevalence across the country. This data was jointly collected by the National Malaria Elimination Programme (NMEP), the National Population Commission (NPC), the National Bureau of Statistics (NBS), and the Malaria Partnership in Nigeria.

### Sampling

The Nigeria National Population and Housing Census conducted in the year 2006 served as the sampling frame for the NMIS 2015 study. Each of the 36 states in Nigeria was classified into Local government areas (LGAs), and each LGAs into localities. In each locality were also enumeration areas (EAs) or clusters which formed the primary sampling unit. Selection of study participants was in two stages. In the first stage, a total of 333 EAs (comprising 9 EAs per state, including the FCT) were randomly selected. Twenty-five households in each EAs that were selected by equal probability systematic sampling represents the second stage of sampling. All women who were between 15 and 49 years of age, residing permanently in the households or visitors who were present on the night prior the start of the survey were enlisted. The sample size was calculated in such a way that 1338 women in each of the six geopolitical zones participated in the study. The full description of field procedures are well stated in the full report of the surveys [28].

### Data collection procedure

Data collection took place between October and November 2015. Questionnaire was used to obtain data about malaria prevention activities that the women practice and blood samples were taken from children 6 to 59 months to screen for anaemia and malaria parasite [28].

### Laboratory procedures

#### Rapid diagnostic test for malaria

A drop of blood from the finger/heel prick was also used for malaria rapid diagnostic test (RDT) using SD BIOLINE Malaria Ag P.f (HRP-II)™ (Standard Diagnostics, Inc.) to detect the histidine-rich protein II antigen of *P. falciparum*. All the field laboratory scientists were trained to perform RDT according to the manufacturer’s instruction. An applicator was used to draw up a small volume of blood which was placed in the well of the testing device and 2 drops of the buffer added to the other well. The result was read after 15 min. Results were then given to the parent/caregiver of the children and also recorded in the biomarker questionnaire. Children with positive RDT were given age-appropriate malaria treatment according to the Nigeria national malaria treatment guidelines as long as they were not currently on treatment for malaria and had not had such treatment 2 weeks prior the survey [28].

#### Malaria diagnosis by microscopy

Both thick and thin films were prepared on the field using slides. Each slide was given a bar code label which was recorded in each biomarker questionnaire and the blood sample transmittal form that was used to track the blood samples from the point of collection to the laboratory. The slides were dried and transported in slide boxes. Thin smears were fixed using absolute methanol at the end of each day. The slides were then stained and read. Thick smears were examined first to determine the presence of malaria parasite. The thin smears for all positive thick smears were then examined to determine the species of malaria parasite that was present [28].

#### Testing for anaemia

Trained health technicians collected blood samples from the children using a finger or heel pricks that were carried out with a single use retractable, spring loaded, sterile lancet. Blood was then collected in a microcuvette. A battery operated portable HemoCue^®^ analyser was used for haemoglobin analysis on site and results were obtained within 1 min. The results were recorded in the biomarker questionnaire. Both verbal and written results were then given to the child’s parents/caregiver. Parents whose children had haemoglobin less than 8 g/dl were counselled and given referral letter to a health facility for proper care and the household was given a brochure showing the causes and prevention of anaemia [28].

#### Description of variables

The dependent variable was the outcome of the malaria tests that was determined using RDT and microscopy. Due to wide discrepancies between the outcomes of the two tests, data was analysed along the two outcomes.

The main independent variable was housing type that was classified as either improved or unimproved based on the materials with which the floor, roof, and wall were built. The flooring materials were categorized into either “Improved materials” consisting of cement, ceramic tiles, vinyl asphalt strips, parquet and polished wood or “Unimproved materials” which were made up of earth, sand, dung, rudimentary, wood planks, palm, bamboo, and others [29, 30].

The wall materials were categorized as either “Improved materials” (cement, stone with lime/cement, cement blocks and bricks) or “Unimproved materials” (no wall, cane/palm/trunks, dirt, rudimentary, bamboo with mud, stone with mud, uncovered adobe, plywood, and others. Roofing materials were categorized into “Improved materials” (Cement and roofing shingles) and “Unimproved Materials” (no roof, thatch/palm leaf, sod, rudimentary, rustic mat, palm/bamboo, wood planks, cardboard, wood, and others) [29, 30]. All the building materials were then categorized as (i) totally improved-wherein the roofing, wall and floor materials were all improved, (ii) Partially improved-wherein only one or two of the roofing, wall and floor materials were all improved and (iii) Unimproved-wherein none of the roofing, wall and floor materials was improved.

Other independent variables include household wealth index, age, and sex of children, mother’s education, place of residence, region, location, sleeping under long-lasting insecticide-treated net or any ever treated nets, fever in last 2 weeks, and having anaemia. The ages of under-five children were categorized into 0–6, 7–23, and 24–59 months as used in a study by Carvajal-Vélez et al. [31].

#### Data analysis

Data were analysed using Stata version 14. Basic descriptive statistics were used in describing the distribution of children and the malaria prevalence by the independent variables (flooring materials, wall materials, roofing materials, all building materials, wealth index, age, sex, mother’s education, place of residence, region, location, sleeping under long-lasting insecticide-treated net or any ever treated nets, having fever in last 2 weeks, and having anaemia). Logistic regression models were used in identifying the risk factors of malaria among the children. Bivariate logistic analysis was conducted in order to identify individual candidate variables. All significant variables at bivariate level (p-value less than 0.10) were included in the multiple logistic regression as shown in Tables 5, 6. The presence of other variables were controlled by accounting for confounders to obtain the adjusted odds ratio in the two Tables. At the level of multivariate analysis, three models were used to define the relationship between malaria prevalence and housing type. Model 1 is the bivariate model that examined the relationship between housing type and prevalence of malaria while model 2 included the dependent variable, housing type, and the use of treated nets. Model 3 comprised the dependent variable, housing type, use of treated nets, and other independent variables. Data were weighted and significance was evaluated at 5%.

## Results

In all, a total of 6991 children were captured in the survey among which 5753 and 6025 were tested for malaria parasite using the RDT and microscopy methods, respectively. Table 1 shows the distribution of U5 children tested for malaria parasite using RDT and Microscopy. The percentage of children positive for malaria increased with increase in age for both RDT and microscopy tests (p < 0.001), and most prevalent among male children. A greater proportion of U5 children who were malaria positive for RDT and microscopy reside in rural areas, and in the North west geopolitical zone.

**Table 1:** Distribution of U5 children tested for malaria parasite using RDT or Microscopy and association between test outcomes and selected characteristics

Variables	Number of children	%	Malaria test			
			RDT+	*χ*^2^ p-value	Micro+	*χ*^2^ p-value
Child’s age (months)						
0–6	755	10.8	21.6	< 0.001*	10.0	< 0.001*
7–23	1914	27.3	36.2		20.3	
24–59	4342	61.9	49.5		30.9	
Sex						
Male	3574	51.0	46.2	0.063	27.8	0.276
Female	3437	49.0	44.0		26.9	
Zone						
North Central	1309	18.7	50.7	< 0.001*	32.1	< 0.001*
North East	983	14.0	42.9		26.5	
North West	2286	32.6	58.2		37.1	
South East	601	8.6	31.7		13.9	
South South	780	11.1	28.9		19.4	
South West	1053	15.0	32.1		15.3	
Location						
Urban	2350	33.5	24.1	< 0.001*	11.4	< 0.001*
Rural	4661	66.5	55.7		35.6	
Usual resident						
No	69	1.0	36.8	0.147	25.2	0.598
Yes	6942	99.0	45.2		27.4	
Wealth quintile						
Poorest	1474	21.0	64.1	< 0.001*	43.1	< 0.001*
Poorer	1612	23.0	62.7		41.0	
Middle	1333	19.0	49.2		27.7	
Richer	1289	18.4	30.2		16.8	
Richest	1303	18.6	12.7		4.3	
Slept under ever treated net						
No	3963	56.5	42.1	< 0.001*	25.7	< 0.001*
Yes	3048	43.5	49.0		29.5	
Slept under LLITNs						
No	3980	56.8	42.0	< 0.001*	25.7	< 0.001*
Yes	3 031	43.2	49.1		29.5	
Dwelling sprayed against mosquito in last 12 months						
No	6867	98.6	43.9	0.006*	27.3	0.111
Yes	95	1.4	29.9		20.0	
Fever in last 2 weeks						
No	4123	59.0	37.4	< 0.001*	23.7	< 0.001*
Yes	2868	41.0	55.1		32.1	

RDT+: positive on rapid diagnostic test, Micro+: positive on microscopy test

* Significant at 5% *χ*^2^ test

Moreover, malaria prevalence reduces with increasing wealth quintile and place of residence (p < 0.001). For instance, children who belonged to the richest wealth quintile had a lesser percentage of malaria positives (RDT+: 12.7%, Micro+: 4.3%) compared to those in the poorest wealth quintile (RDT+: 64.1%, Micro+: 43.1%). Malaria was more prevalent among LLIN users (RDT+: 29.5%, Micro+: 49.1%) as well as those that used any ever-treated nets (RDT+: 29.5%, Micro+: 49.0%). Also, a greater proportion of children whose mothers reported no symptoms of fever in past 2 weeks prior the survey tested positive for malaria parasite on RDT (32.1%) and microscopy (55.1%). The most prevalent species of malaria parasite identified from blood film microscopy among U5 children was *P. falciparum* (93.6%). Other isolated species include *Plasmodium ovale* (5.1%) and *Plasmodium malariae* (1.3%).

Table 2 shows the diagnostic performance of an RDT compared with the microscopy test for malaria diagnosis. Using the microscopy test as a gold standard, RDT was more sensitive than being specific (87.6% vs 75.8%).

**Table 2:** Relationship between the results of the RDT and microscopy tests

	Number tested	RDT test			*χ*^2^ p-value
		Positive	Negative	Total	
Number tested		2387	3366	5753**	
Microscopy test					
Positive	1572	87.6	12.4	27.3	< 0.001
Negative	4181	24.2	75.8	72.7	
Total	5753**	41.5	58.5		

The HRP-II that was used for RDT can only detect. *P. falciparum*. The *P. ovale* and *P. malariae* which were found on microscopy also account for the difference between the two tests

RDT+: positive on rapid diagnostic test, Micro+: positive on microscopy test

* Significant at 5% *χ*^2^ test

** Included only the children that have results for both tests

Anaemia was significantly associated with the presence of malaria parasite. Children who were malaria positive showed a higher prevalence of severe anaemia on RDT+ (87.6%) and Micro+ (67.4%) than those who were not anaemic (RDT+ = 31.6%, Micro+ = 12.9%) (Table 3).

**Table 3:** Prevalence of anaemia among malaria positive children

Anaemia level	Number tested	%	RDT+	p-value	Micro+	*χ*^2^ p-value
Severe	224	3.7	87.6	< 0.001*	67.4	< 0.001*
Moderate	2376	39.4	61.9		40.6	
Mild	1524	25.3	37.5		20.6	
Not anaemic	1905	31.6	25.1		12.9	

RDT+: positive on rapid diagnostic test, Micro+: positive on microscopy test

* Significant at 5% *χ*^2^ test

The risk of malaria positive RDT and microscopy was significantly lower for children living in houses built with improved floor materials (RDT 35.3% vs Micro 19.2%, p < 0.001) compared to those living in houses built with unimproved floor materials (RDT 56.4% vs Micro 36.9%, p < 0.001) (Table 4).

**Table 4:** The relationship between housing characteristics and malaria infection among under-five children in Nigeria

Housing characteristics	N	%	Malaria test			
			RDT+	p-value	Micro+	*χ*^2^ p-value
Floor materials						
Unimproved	3283	46.8	56.4	< 0.001*	36.9	< 0.001*
Improved	3728	53.2	35.3		19.2	
Roof materials						
Unimproved	1889	26.9	59.7	< 0.001*	42.8	< 0.001*
Improved	5122	73.1	39.7		21.7	
Wall materials						
Unimproved	3034	43.3	61.4	< 0.001*	39.9	< 0.001*
Improved	3977	56.7	32.9		18.2	
All building materials						
Totally improved	2969	42.4	32.4	< 0.001*	17.0	< 0.001*
Partially improved	2630	37.5	49.4		28.9	
Nothing improved	1412	20.1	64.2		47.1	
Total	7011	100.0	45.1		27.3	

RDT+: positive on rapid diagnostic test, Micro+: positive on microscopy test

* Significant at 5% *χ*^2^ test

This similar pattern was observed for children living in houses built with an improved roof and wall materials. The results further show that children who lived in totally improved houses (RDT 32.4% vs Micro 17.0%, p < 0.001) experienced lesser incidence of malaria infection compared to those who lived in houses built with non-improved housing materials (RDT 64.2% vs Micro 47.1%, p < 0.001).

### Bivariate results

Table 5 showed the unadjusted and adjusted odds ratio for the relationship between malaria infection from microscopy, socio-demographic characteristics, and types of housing materials. At the unadjusted bivariate level of analysis, the odds of malaria infection were higher among children age 24–59 months (OR = 4.49, CI 2.06–9.79), the North West region of Nigeria (OR = 3.46, CI 2.78–4.32), in rural areas (OR = 3.98, CI 3.42–4.63), in households with the poorest wealth quintile (OR = 15.61, CI 11.57–21.06), slept under ever-treated net (OR = 1.31, CI 1.18–1.45) and LLIN (OR = 1.32, CI 1.19–1.46). Furthermore, the quality of housing materials versus living in houses with unimproved floor materials (OR = 2.50, CI 2.22–2.82), unimproved roof (OR = 2.92, CI 2.57–3.33) and unimproved wall materials (OR = 3.05, CI 2.70–3.44), and all non-improved building materials (OR = 4.77, CI 4.06–5.61) were significantly associated with higher risks of malaria infection among U5 children. Indoor spraying of the household was protective against malaria infection.

**Table 5:** Unadjusted and adjusted odds ratio of factors influencing malaria infection using microscopy test results

Characteristics	Unadjusted estimates			Adjusted estimates		
	OR	95% CI	p-value	aOR	95% CI	p-value
Child age (months)						
0–6	1	–		1	–	
7–23	2.58	1.18–5.67*	0.02	2.29	1.21–4.34*	< 0.01
24–59	4.49	2.06–9.79*	< 0.01	5.67	3.01–10.70*	< 0.01
Zone						
South West	1	–		1	–	
North Central	2.02	1.60–2.55*	< 0.01	0.61	0.47–0.79*	< 0.01
North East	1.79	1.41–2.27*	< 0.01	0.35	0.27–0.46*	< 0.01
North West	3.46	2.78–4.32*	< 0.01	0.49	0.37–0.64*	< 0.01
South East	0.86	0.63–1.16*	0.33	0.59	0.44–0.79*	< 0.01
South South	1.28	0.99–1.67*	0.06	0.42	0.31–0.55*	< 0.01
Location						
Urban	1	–		1	–	
Rural	3.98	3.42–4.63*	< 0.01	1.59	1.33–1.89*	< 0.01
Wealth quintile						
Richest	1	–		1	–	
Poorest	15.61	11.57–21.06*	< 0.01	5.51	3.83–7.93*	< 0.01
Poorer	13.51	10.05–18.16*	< 0.01	5.15	3.72–7.13*	< 0.01
Middle	7.06	5.22–9.55*	< 0.01	3.51	2.64–4.65*	< 0.01
Richer	3.78	2.77–5.1*8	< 0.01	1.89	1.46–2.45*	< 0.01
Slept under ever treated net						
No	1	–		1	–	
Yes	1.31	1.18–1.45*	< 0.01	1.21	1.08–1.36*	< 0.01
Slept under LLIN						
No	1	–		1	–	
Yes	1.32	1.19–1.46*	<0.01	1.21	1.08–1.36*	< 0.01
Dwelling sprayed against mosquito in last 12 months						
Yes	1	–		1	–	
No	1.83	1.19–2.85*	< 0.01	0.37	0.10–1.38	0.14
Floor materials						
Improved	1	–		1**	–	
Unimproved	2.50	2.22–2.82*	< 0.01			
Roof materials						
Improved	1	–		1**	–	
Unimproved	2.92	2.57–3.33*	< 0.01			
Wall materials						
Improved	1	–		1**	–	
Unimproved	3.05	2.70–3.44*	< 0.01			
All building material						
Totally improved	1	–		1	–	
Partially improved	2.21	1.93–2.54*	< 0.01	1.02	0.86–1.21	0.82
Nothing improved	4.77	4.06–5.61*	< 0.01	1.05	1.02–1.12*	0.04

* Significant at 5% test of logistic regression; ** dropped due to multicollinearity:* OR* odds ratio,* aOR* adjusted odds ratio

The adjusted odds ratio for socio-demographic characteristics and types of housing materials associated with malaria symptoms showed that their pattern of association with childhood malaria infection remained virtually the same as when the factors were unadjusted for. However, living in the South West was associated with lower odds of malaria infection among U5 children compared with living in the North Central, North East, North West, South East, and South–South regions.

### Multivariate results

The results in Table 6 shows the adjusted odd ratio for the factors associated with the prevalence of malaria infection (determined by microscopy) among U5 children. There is a significantly-reduced likelihood of malaria infection among U5 if living in an improved housing. In the first model, U5 children who lived in houses built with non-improved materials had higher adjusted odds of having malaria infection (aOR = 4.8, CI 4.06–5.61, p < 0.001) than those living in houses built with completely improved materials. This significant association seen in model I was also observed in model II (aOR = 4.7, CI 4.01–5.57, p < 0.001). Thus, indicating that indoor residual spraying, sleeping under an ever treated net and or an LLIN does not have an impact on the significant relationship between living in houses built with completely unimproved materials and contracting malaria. Moreover, controlling for other independent variables greatly reduced the odds of malaria infection when living in a “Nothing improved” housing (aOR = 1.4, CI 1.08–1.80, p = 0.01) observed in model III though the odds ratio was still greater than 1.00.

**Table 6:** Adjusted risk factors for malaria among U5 children based on the microscopic test

Characteristics	Model I		Model II		Model III	
	OR (95% CI)	p-value	aOR (95% CI)	p-value	aOR (95% CI)	p-value
All building materials						
Totally improved	1	–				
Partially improved	2.21 (1.93–2.54)	0.00*	2.21 (1.92–2.54)	0.00*	1.03 (0.85–1.25)	0.75
Nothing improved	4.77 (4.06–5.61)	0.00*	4.73 (4.01–5.57)	0.00*	1.39 (1.08–1.80)	0.01*
Slept under an ever treated net						
No			1	–	1	–
Yes			1.16 (0.43–3.15)	0.77	0.74 (0.17–3.18)	0.69
Slept under LLIN						
No			1	–	1	–
Yes			0.90 (0.33–2.45)	0.84	1.11 (0.26–4.79)	0.89
Indoor residual spray						
Yes			1	–	1	–
No			1.24 (0.74–2.08)	0.42	1.21 (0.64–2.31)	0.56
Child age (months)						
0–6					1	–
7–23					2.17 (0.95–4.97)	0.07*
24–59					4.84 (2.13–10.99)	0.00*
Zone						
South West					1	–
North Central					0.65 (0.47–0.89)	0.01*
North East					0.48 (0.35–0.67)	0.00*
North West					0.69 (0.50–0.96)	0.03*
South East					0.51 (0.35–0.75)	0.00*
South–South					0.70 (0.50–0.98)	0.04*
Location						
Urban					1	–
Rural					1.54 (1.25–1.91)	0.00*

*OR* odds ratio *aOR* adjusted odds ratio

* Significant at 5% test of logistic regression coefficients significance

Other identified predictors of malaria infection include child’s age (24–59 months) and the location of residence. The adjusted odds of malaria infection was about 5 times more likely in older children age 24–59 months (aOR = 4.8, CI 2.13–10.99, p < 0.001), and about two times (aOR = 1.5, CI 1.25–1.91, p < 0.001) higher in rural areas. However, the likelihood of malaria infection was 0.48 (CI = 0.35–0.67, p < 0.001) lower in the North East than the South West regions.

## Discussion

Considering the contribution of malaria to under-five morbidities and mortalities in Nigeria, this study is important as it assessed how housing types were related to malaria infection among U5s in Nigeria. This gives an insight into how housing type can contribute to efforts at controlling malaria among U5 children in Nigeria.

Overall, children living in partially improved and non-improved housing consistently were more likely to have malaria infection compared with those living in improved housing. This likelihood persisted even after adjustment for sleeping under LLINs and indoor spraying which are among the common activities that Nigerians employ in the control of malaria. An earlier study from Uganda did not consider these other control strategies for malaria in their analysis [32]. Our findings gave credence to the idea that better protection from malaria can be achieved by building houses with modern materials in addition to the use of ITN and indoor spraying. Combination of these strategies can create better means of preventing malaria with exponential effect among U5 children in Nigeria and this can improve the overall infant and U5 morbidity and mortality in the country.

However, following further adjustment for age, region, location, mothers’ education and wealth quintile, only non-improved housing still predicted malaria infection among U5. This shows the strong relationship between non-improved housing and malaria infection among U5 and thus makes a strong case for the need to enforce improved housing as a means of controlling malaria among U5 in Nigeria. By tapping into the economic and cultural transition that is ongoing in Africa now, it will be an excellent opportunity to use this new means of malaria prevention to augment existing strategies and improve malaria control among U5 s [24].

The tendency of the older children to have malaria infection compared with the younger ones is likely to be as a result of the maternal antibodies to malaria which was transferred during pregnancy. These antibodies are mainly immunoglobulin G whose half-life is 6 months [33]. Since the active transfer of the antibody occurs all through pregnancy, the nadir occurs at 6 months of life which explains the reason why the first malaria in children is usually around 6 months of age [34]. The other likely reason for the differential malaria infection rate is the preference of the anopheline mosquitos to bite older children and adults and not infants [35]. The younger infants are also more likely to be breastfeeding and may have the protection of lactoferrin and secretory IgA which have been shown to inhibit malaria parasite growth in vitro [36]. Another reason may also be that the older children will be able to crawl or walk around with less dependence on their mothers, and so, will likely benefit less from the malaria protective behaviours of their mothers. The mothers are more likely to cover their babies’ skin with clothing and also wade off or kill the mosquitoes with bare hands. These protective activities will be less available for older children who are no longer so dependent on their mothers. The male children had more malaria infection compared to the females, although the difference was not significant. This may be as a result of the adventurous nature of the males who are more likely to stay more outdoors and as a result, have more mosquito bites.

Anaemia was significantly associated with the presence of malaria parasite which was a consistent finding with earlier studies [37, 38]. Anaemia has been shown to impair growth and cognitive functions among school children [39, 40], and in extreme cases, it can result in complicated malaria and death [39]. It is frightening that these children were at home with no form of treatment given to these complications of malaria. The possibility of dying from this complication is high among U5s [39] and this is likely an unnoticed group of children who can potentially contribute to U5 mortality in Nigeria. Public enlightenment targeted at parent could address this and also training of patent medicine sellers from whom many parents seek malaria treatment for their wards.

The inverse relationship between U5 malaria infection and wealth quintile is not surprising as people with higher wealth quintile were more likely to be able to afford improved houses and more likely to be educated and have better access to knowledge about steps to prevent malaria infection [21, 41]. They were also more likely to be able to afford ITN and be able to use them correctly as well as be able to afford insecticides used for indoor spraying. In addition, people in the higher wealth quintile are more likely to be found in urban areas.

The rural dwellers, on the other hand, were more likely to have factors that promote malaria transmission generally aside from non-improved housing, like bushes and inappropriate waste disposal (which present excellent breeding spaces for mosquitos), reduced access to ITN, indoor sprays and lack of knowledge of steps to prevent malaria infection. Persons living in urban environment have greater likelihood of having improved houses, higher socioeconomic status and limited number of breeding sites [42] and thus, lower likelihood of malaria infection. However, urban malaria continues to be a concern globally since it accounts for 6–28% of the projected annual malaria incidence [43].

Children resident in South west Nigeria were more likely to have malaria infection than children from the other parts. Different factors have been described to influence the prevalence, distribution, and transmission of malaria vectors as it relates to recent changes in climatic and environmental factors [44].

In this study, children who ever used ITN or were current users of ITN were more likely to have a malaria infection. This finding is of serious concerns because it contravenes the expectation that the use of ITN will protect them from malaria. This is probably due to the fact that children were only put to net-use after they might have been exposed to malaria or possibly because of living in malaria-prone areas. Other possible reasons why malaria is more prevalent among ITN users are diminishing bioefficacy of the nets, lowered susceptibility of the mosquitoes to pyrethroid insecticides, wrong use of the ITNs, use of ITNs with holes, and the habit of sleeping on the couch in the living room before sleeping under a treated net [45]. This calls for a holistic approach to malaria prevention like indoor residual spraying, living in an improved housing and maintaining a clean environment.

Some limitations were identified in this study. It was not clear if the children with features of severe malaria were already on antimalarial drugs when they were seen so we were not sure the parents identified these complications and sought for help before the study identified them. The differences in the specificity and sensitivity of the diagnostic methods (RDT and microscopy) and how sampling strategy was conducted which may be biased to certain people in the population are worthy of mention.

## Conclusion

Housing type is an essential risk factor for malaria occurrence among U5 children in Nigeria. Non-improved housing predicted malaria infection among U5 in this study. Also, older children, those living in rural areas and children from the poorest households were most affected. Improved housing is a promising means to support a more integrated and sustainable approach to malaria prevention, and ending the incidence of malaria as stipulated in the Nigeria national malaria policy and target 3.3 of Goal 3 of the sustainable development goals. Education of the Nigerian people on the malaria protection role of modern housing and empowerment to encourage the adoption of modern house design in addition to other malaria preventive strategies can help to augment the current malaria control measures among U5 children.

## Abbreviations

U5: under-five
RDT: rapid diagnostic test
OR: odds ratio
CI: confidence interval
NDHS: Nigeria Demographic and Health Surveys
EAs: enumeration areas
LLINs: long-lasting insecticide-treated nets
IRS: indoor residual spraying
ACT: artemisinin-based combination therapy
IPTp: intermittent preventive therapy for pregnant women
RBM: Roll Back Malaria
AIM: Action and Investment to Defeat Malaria
FCT: Federal Capital Territory
NMIS: Nigeria Malaria Indicator Survey
NMEP: National Malaria Elimination Programme
NPC: National Population Commission
NBS: National Bureau of Statistics
LGAs: Local Government Areas
Micro+: positive on microscopy
ITN: insecticide-treated net
